# Adjuvant Chemotherapy in Patients with Locally Advanced Upper Tract Urothelial Carcinoma with or without Kidney Transplantation

**DOI:** 10.3390/jcm13071831

**Published:** 2024-03-22

**Authors:** Nai-Wen Chang, Yu-Hui Huang, Wen-Wei Sung, Sung-Lang Chen

**Affiliations:** 1 Department of Urology, Chung Shan Medical University Hospital, Taichung 402, Taiwan; catastream@gmail.com (N.-W.C.); flutewayne@gmail.com (W.-W.S.); 2 Department of Physical Medicine and Rehabilitation, Chung Shan Medical University Hospital, Taichung 402, Taiwan; 3 School of Medicine, Chung Shan Medical University, Taichung 402, Taiwan

**Keywords:** adjuvant chemotherapy, kidney transplantation, urothelial carcinoma, survival rate

## Abstract

**Background**: The incidence of upper tract urothelial carcinoma (UTUC) is uniquely high in kidney transplant (KT) recipients in Taiwan. The evidence of adjuvant chemotherapy (AC) in UTUC is contradictory. We have sought to determine whether AC is associated with potential benefits related to locally advanced UTUC after KT. **Methods**: We retrospectively analyzed 134 patients with locally advanced UTUC (at least stage T2) and patients who were administrated AC after unilateral or bilateral nephroureterectomy with bladder cuff excision. Of these 134 patients, 57 patients fulfilled our inclusion criteria. We used 23 KT and 34 non-KT locally advanced UTUC patients for comparison. **Results**: The mean follow-up time was 52.35 ± 34.56 and 64.71 ± 42.29 months for the KT and non-KT groups, respectively. The five-year disease-free survival (DFS) and overall survival (OS) rates were 45.7% vs. 70.2% and 62.8% vs. 77.6%, for the KT and non-KT groups. The Kaplan–Meier curve and the log rank test revealed significant differences in the DFS and OS rates between the two groups, *p* = 0.015 and 0.036. The influence of chemotherapy on graft kidney function was mild. Only three in the KT group and two in the non-KT group developed > grade 2 nephrotoxicity. **Conclusions**: Our study suggested that KT patients with locally advanced UTUC who had been administered AC after surgery presented worse OS and DFS than non-KT patients. KT patients tolerated the AC course well, and their nephrotoxicity levels were mild and acceptable.

## 1. Introduction

In Taiwan, upper tract urothelial carcinoma (UTUC) is more common than renal cell carcinoma, accounting for approximately 30% to 40% of all cases of urothelial carcinoma (UC) [1]. In addition to aristolochic acid consumption and the endemic nature of black foot disease, kidney transplant (KT) is also one of the leading risk factors for UTUC in Taiwan [2]. Our previous studies found that KT recipients have a higher incidence of bilateral UTUC and demonstrated that KT patients with UTUC present aggressive pathological features, with a high proportion exhibiting advanced-stage and high-grade features [3,4].

The inherent differences in the molecular and anatomical characteristics between the upper tract and the urinary bladder produce a discordance in UC treatment effects. Previously, systemic treatment had not been proven to induce benefits for locally advanced UTUC. The evidence obtained from adjuvant chemotherapy (AC) use in relation to UTUC indicated contradictory results, owing to the studies’ retrospective nature and small sample sizes [5]. Furthermore, the chemotherapy regimens currently used for UTUC are the same as those offered for bladder UC, which is associated with more robust and high-level evidence.

Nephroureterectomy and bladder cuff excision, followed by surveillance, have remained the treatment of choice for localized UTUC [6]. Recently, the POUT trial demonstrated that AC significantly improved disease-free survival (DFS) at a median follow-up of 30.3 months for UTUC [7]. However, AC studies concerning UTUC in KT patients, characterized by multiple-site occurrence and high invasiveness, are sparse. However, the Checkmate 274 trial proved that adjuvant immunotherapy extends DFS in patients with high-risk muscle-invasive urothelial carcinoma after radical surgery [8]. By maintaining immunosuppression to keep graft function and augmenting immunity to achieve the optimal cancer therapeutic effects at the same time, immunotherapy remains difficult in KT patients with UTUC [9]. Therefore, chemotherapy is still the standard adjuvant treatment for KT patients.

A population-based study revealed improved overall survival (OS) rates in pT3/T4 and/or pN+ patients (*n* = 3253) [10], while a multicenter cohort study did not indicate any improvements in pT2–T4 and/or pN+ patients (*n* = 1544) [11]. Additionally, only two reports have demonstrated that gemcitabine plus cisplatin (GC) led to significant OS and DFS rate benefits related to bladder UC and UTUC in KT patients [12,13]. Research has indicated that the immunosuppressive status of KT recipients is still associated with more malignant UTUC behavior in KT patients [14]. Due to the high potential of local tissue invasion and distant metastasis in KT patients with UTUC, we analyzed cases with locally advanced UTUC (at least stage T2) and that were administrated AC after operation. Their characteristics and survival outcomes were compared with those with UTUC who had received AC without KT. We also sought to understand the effects and safety of AC in relation to UTUC with or without KT.

## 2. Materials and Methods

This retrospective cohort study was approved by the Institutional Review Board of Chung Shan Medical University Hospital (IRB number: CS1-23024). We enrolled patients who had undergone open-method or laparoscopic unilateral or bilateral nephroureterectomy with bladder cuff excision for UTUC between January 2011 and December 2021 in our hospital. In total, 134 patients with locally advanced UTUC (stage at least T2) were recruited for this study. All participants had satisfactory hematological and biochemical blood profiles and a glomerular filtration rate (GFR) of 30 mL/min or higher. The patients who had received immunotherapy as a first-line adjuvant therapy or who were ineligible for AC were excluded. The remaining 57 patients underwent AC and were divided into two groups, namely KT or non-KT.

A UTUC diagnosis was confirmed via a peer-reviewed pathological examination, and specimens were obtained through an initial URS biopsy or subsequent nephroureterectomy. We checked for concurrent urinary bladder UC via preoperative cystoscopy. The postoperative follow-up protocol comprised abdominal +/− chest compacted tomography (CT), urine cytology, and cystoscopy.

### 2.1. Chemotherapy Regimen and Schedule

The AC group was administered gemcitabine intravenously at 800 mg/m^2^ on days 1, 8, and 15; they were then administered cisplatin at 70 mg/m^2^ intravenously on day 2. The cycles were repeated every 28 days. The hematologic and non-hematologic toxicities were graded according to the Common Terminology Criteria for Adverse Events (CTCAE). Patients with renal function impairment after nephroureterectomy and bladder cuff excision (eGFR < 60 mL/min) were allowed to use carboplatin (area under curve: 4.5) rather than cisplatin. The hematologic toxicities included neutropenia, anemia, and thrombocytopenia; the non-hematologic toxicities included nausea/vomiting, nephrotoxicity, hepatotoxicity, and skin rash. Recombinant human granulocyte colony stimulating factor was administered to treat hematologic toxicities when patients developed high-grade toxicities. The immunosuppressant dosage remained unchanged during the chemotherapy course.

### 2.2. Outcome Measures

The DFS was defined as the time from the surgery until the first recurrence in the tumor bed or metastasis or until death from any cause. The OS was defined as the time from the surgery to death from any cause. A second primary cancer, including muscle-invasive bladder cancer and contralateral UTUC, was regarded as an event to censor. On the other hand, non-muscle-invasive bladder cancer was not regarded as an event to censor.

### 2.3. Statistical Analysis

Continuous data were analyzed using *t* tests, and chi-square tests were used to compare the categorical variables in the different groups. A univariate analysis was conducted, and significant risk factors from the univariate analysis were used in a multivariate analysis. A multivariate logistic regression analysis was used to determine the factors that were significantly associated with UTUC. Kaplan–Meier curves and log-rank tests were used to compare the overall survival and to conduct a disease-free survival analysis. A statistical analysis was performed using SPSS (SPSS for Windows Version 25.0, SPSS Inc., Chicago, IL, USA). A two-tailed *p*-value of < 0.05 was regarded as statistically significant.

## 3. Results

Among 134 patients, 57 patients were pathologically proven to have at least stage T2 UTUC and underwent AC; 23 KT and 34 non-KT UTUC patients were used for comparison, respectively. Between the two groups, no statistically significant differences were found in terms of age, the pathological stage of T and N, surgical positive margin, histological grading, and lymphovascular invasion. However, there were more female patients and multifocal tumors, a higher carcinoma in situ (CIS) proportion, and smaller tumor sizes in the KT group than in the non-KT group (all *p* < 0.05). There were significantly more concurrent contralateral UTUC, and synchronous tumors located in the renal pelvis and ureter in the KT group than in the non-KT group. We observed more concurrent bladder urothelial carcinoma in the KT group (*p* = 0.076). The mean follow-up times in the two groups were 52.35 ± 34.56 and 64.71 ± 42.29 months in the KT and non-KT groups, respectively. The amount of unilateral or bilateral nephroureterectomy and bladder cuff excision underwent via the open method was significantly higher in the KT group (*p* = 0.02). Synchronous renal pelvis and ureter UC lesions were more common in the KT group (*p* = 0.007) (Table 1).

Regarding the AC regimen, there were 13 patients with gemcitabine + cisplatin (G + C) and 10 with gemcitabine + carboplatin (G + Carbo) in the KT group. There were 18 with G + C; 11 with G + Carbo; 2 with gemcitabine only; 2 with methotrexate, vinblastine, doxorubicin, and cisplatin (MVAC); and 1 with an unknown formula in the non-KT group. Most of the patients (44/57) were able to tolerate chemotherapy for three to six cycles, but 11 patients were unable to tolerate it, and they only received one to two cycles (Table 1).

**Figure 1:**
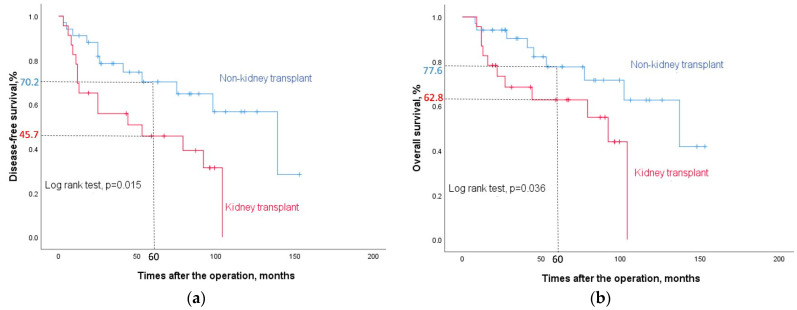
(**a**) Disease-free survival after the operation. The 5-year disease-free survival rates are 45.7% and 70.2% in the KT and non-KT group, respectively. (**b**) Overall survival after the operation. The 5-year overall survival rates are 62.8% and 77.6% in the KT and non-KT group, respectively.

DFS and OS rates were statistically favored in the non-KT group. The five-year DFS and OS rates were 45.7% vs. 70.2% and 62.8% vs. 77.6%, respectively, between the KT and non-KT groups. The Kaplan–Meier curve and the log-rank test revealed significant differences in the DFS and OS rates between the KT and non-KT groups, namely, *p* = 0.015 and 0.036, respectively (Figure 1a,b).

The univariate and multivariate analyses with the Cox proportional hazards regression model revealed that the open surgery method is an independently unfavorable prognostic factor for DFS rates, and tumors located in the renal pelvis are an independently favorable prognostic factor for OS, respectively. However, KT and positive surgical margins were associated with statistically significant differences in univariate but not multivariate analyses during the DFS and OS rate evaluations (Table 2).

The hematologic toxicity items were statistically unfavorable in the KT group. There was no difference in the non-hematologic toxicities between the two groups (Table 3).

## 4. Discussion

In Taiwan, many studies have reported an unusually high prevalence and predominance of UTUC among females [3,4,15]. Vague symptoms and the delayed diagnosis of UTUC mean that tumors are often muscle-invasive or locally advanced at presentation, resulting in poorer survival outcomes compared to UC of the urinary bladder. Indeed, oncological outcomes after surgery for UTUC have not changed in the last two decades [16]. The tumor grade and stage at nephroureterectomy and bladder cuff excision have played a key role in overall survival rates [17]. Unlike neoadjuvant chemotherapy for invasive bladder UC, accurately predicting preoperative high-risk or organ-confined disease in UTUC remains difficult. Neoadjuvant chemotherapy for UTUC is most likely characterized by either over- or under-treatment with definite risk stratification [18]. Conversely, AC for UTUC may be dependent upon the ability to administer full-dose cisplatin with curative intent when patients still have a functioning kidney after a nephroureterectomy. However, the evidence obtained from AC use in UTUC has indicated contradictory OS results due to the retrospective nature of such studies [5]. The recent POUT trial recruited patients with UTUC after a nephroureterectomy staged at either pT2–T4 pN0–N3 M0 or pTany N1–3 M0. This randomized control trial significantly improved DFS rates in patients with locally advanced UTUC, and adjuvant platinum-based chemotherapy should be considered a new standard of care after nephroureterectomy [7]. However, it is rarely used in KT recipients due to platinum’s nephrotoxicity and the synergistic effects of chemotherapy and immunosuppressants. The safety of platinum-based chemotherapy for KT recipients has been demonstrated in previous studies [13], but its effects on the prognoses of patients with stage ≥ T2 disease remain unclear.

The immunosuppressive status of KT recipients may be associated with aggressive pathological features of UTUC. A previous cohort study revealed post-KT patients with 60% stage ≥ pT2 and 83% high-grade UC [3]. Another propensity-matched study also indicated a worse oncological outcome of UTUC in end-stage renal disease (ESRD) patients after KT compared to ESRD without KT [14]. However, the study did not mention the adjuvant treatment after the operation. Regarding the nephrotoxicity of chemotherapeutic regimens (particularly cisplatin-based), AC for KT patients with UTUC is more cautious and conservative. One previous study enrolled seven KT patients diagnosed with locally advanced UC (2 UTUC+ UBUC, 4 UTUC, 1 UBUC) who were treated with a pre- and postoperative GC AC. The side effects were tolerable and reversible, with minor impacts on graft function [12]. Wang et al. reported 22 KT patients with locally advanced UC. Eleven patients who underwent surgery and received AC were compared with the remaining eleven patients in the surgery-alone group. The results indicated survival benefits in the AC group, with a shorter follow-up time (21 months). Among these eleven patients with advanced UC who underwent AC, only seven patients had UTUC [13]. Our study revealed that the outcomes of AC for KT patients with UTUC were worse in terms of DFS and OS rates. The five-year DFS rates were 45.7% and 70.2% in the KT and non-KT group, respectively. The five-year OS rates were 62.8% and 77.6% in the KT and non-KT group, respectively. Another study focused on the oncologic outcomes of AC for pT3N0M0 [18]. This study demonstrated a five-year DFS rate of 74.4% and a cancer-specific survival (CSS) rate of 80.5% in the AC group. One Korean study enrolled patients with the same criteria for UTUC (≥T2) who underwent AC with the GC or MVAV regimen. The five-year OS and DFS rates were 78.1% and 62.5%, respectively [19]. The OS and DFS rates in our non-KT group were comparable with the above-mentioned series. Although cisplatin-based chemotherapy should be the preferred agent when possible, our results suggest that patients for whom cisplatin is contraindicated because of poor renal function could still derive benefits from the alternative gemcitabine plus carboplatin regimen.

One Asian report stated that bladder UC and UTUC occurred more in KT recipients than in the general population, by a factor of 25.5 and 129.5, respectively [20]. Compared to the non-KT group, more female patients had stage > pT2 UTUC in the KT group based on our study results. In contrast to the epidemiology of UTUC in Western countries, there are more female UTUC patients in Taiwan [21,22,23]. This suggests that risk factors other than smoking might play a significant role in UTUC development in Taiwan [24]. For instance, risk factors such as analgesic abuse, Chinese herbal agents, exposure to arsenic fumes, the use of immunosuppressive agents, and chronic inflammatory status in KT patients contribute to the predominance of UTUC among female patients [4,9,25,26]. Based on a univariate survival analysis, it appears possible that the immunocompromised status of KT patients impacts the survival benefits of AC in long-term follow-ups. The multivariate analysis failed to demonstrate the statistical significance of KT in relation to AC for UTUC, possibly because of small sample sizes (23 and 34 patients in each group). UTUC after KT is a relatively rare malignancy, even in Taiwan. In the future, UTUC after KT should be examined via inter-professional collaborations with more research groups. Positive surgical margins, open operative methods, and tumor locations in the renal pelvis influenced the oncologic outcomes. Our research demonstrated that the tumor’s location in the renal pelvis is the only independent favorable risk factor for OS. Previous meta-analyses have compared ureteral vs. pelvic-calyceal tumors in relation to oncologic outcomes. This report also revealed that the tumor’s location in the ureter was a poor prognostic factor for both CSS and OS rates [27]. However, one study compared the survival rates associated with open and laparoscopic surgery, and the results indicated no differences in DFS between open and laparoscopic surgery [28]. Open surgery was the only independent unfavorable risk factor for DFS in our study. From a technical perspective, much of the difficulty associated with this surgery in KT patients is due to significant scarring around the graft site. The meticulous ureter dissection at the graft kidney site presented a challenge in laparoscopic surgeries. This may explain why our results indicated that UTUC in KT was associated with more open surgery compared to the non-KT group (*p* = 0.02). Frankly speaking, we also tended to favor open surgery for larger tumor sizes and more invasive clinical stages involving positive lymph nodes on preoperative radiologic images. This may have contributed to open surgery being statistically significant in relation to DFS rates (*p* = 0.015) and marginally significant in relation to OS rates (*p* = 0.06). The increasing use of robotic-assisted operations in urologic and general surgeries has recently precipitated several systematic reviews and meta-analyses comparing the outcomes of robotic-assisted and laparoscopic nephroureterectomy. In this approach, the operating site is magnified in three dimensions on a computer screen, providing a superior view during the robotic-assisted operations. Theoretically, patients receiving minimally invasive operations are more likely to receive AC due to faster recovery rates compared with open surgery. The importance of AC in UTUC may be promoted in the new era of minimally invasive surgery.

A previous study in southern Taiwan revealed earlier-onset and more aggressive pathological characteristics, such as CIS and multifocality, in post-KT UTUC patients [14]. Our study also indicated a higher bladder recurrence rate of 43.5% in the KT group, compared to 35.3% in the non-KT group; however, there was no statistical difference. The discrepancy in the AC treatment effect between the KT and non-KT group may be partly attributed to immunosuppressant-induced DNA damage and precluded or delayed DNA repair mechanisms. In addition, KT associated with highly prevalent BK virus infections also facilitated synergistic effects on the extent of UC malignancy [29]. Furthermore, immune surveillance recovery after AC could be impaired or delayed by immunosuppressive agents. Therefore, interactions between immunosuppressants and AC should be accounted for when formulating a therapeutic strategy. Concerning graft function and the possible synergistic toxicities and side effects caused by immunosuppressants and chemotherapy drugs, decreased dosages of AC and/or changes in immunosuppressants to prevent cancer progression or recurrence are common treatment options for KT patients with AC. Some research has suggested the reduction in calcineurin inhibitor (CNI, cyclosporine A, and tacrolimus) levels to the traditional low bounds to prevent cancer progression and recurrence; however, this is likely to increase the rejection risk [30]. Our study protocol remained unchanged, calling for immunosuppressant dosages during chemotherapy courses. The protocol stipulates that patients with eGFR ≥ 60 mL/min after their operation will be given a standard dose of cisplatin (70 mg/m^2^) regardless of their KT or non-KT status. Carboplatin will be used when eGFR < 60 mL/min. Cisplatin should be the preferred chemo-agent when possible. On the other hand, our results suggest that patients for whom cisplatin is contraindicated because of poor renal function could still derive benefits from the alternative gemcitabine plus carboplatin regimen. Although recombinant human granulocyte colony stimulating factor was administered to treat hematologic toxicities, nearly one-half of the KT patients and one-third of the non-KT patients required dose reductions. Clearly, KT groups presented with more hematologic adverse events, which could be explained by the intense reaction between the immunosuppressant regimen and the chemotherapeutic agents. The influence of chemotherapy on graft kidney function was mild. Only three patients in the KT group and two in the non-KT group developed >grade 2 nephrotoxicity, which was reversed after treatment.

Neoadjuvant or adjuvant immunotherapy currently offers survival benefits in advanced and metastatic UC, particularly for cisplatin-ineligible patients with limited alternatives [31]. However, immunotherapy for UC in KT patients may still be a double-edged sword. The T-cells produced after the administration of immunotherapy act against not only tumor antigens but also against donor alloantigens. Ensuring the optimal therapeutic effects and maintaining graft tolerance in renal transplant patients is difficult. Hence, further studies involving renal transplant patients are warranted. Recent data also suggest potential benefits from two new classes of agents, namely fibroblast growth factor receptor (FGFR) inhibitors and antibody drug conjugate (ADC) in patients with locally advanced or metastatic UTUC for whom systemic chemotherapy is not contraindicated. However, the application of these new therapeutic drugs to KT patients with UTUC requires more clinical trials to elucidate the benefits. To our knowledge, this is the first study to compare the oncologic outcomes related to locally advanced UTUC patients who underwent AC, specifically between KT and non-KT patients. There were several limitations of our study. First, it was a nonrandomized, retrospective, single-center cohort study of Taiwanese patients. The impact of ethnicity on the distribution of metastasis in patients with UTUC has already been explored [32]. Thus, further inferences may not be representative of the general population. However, KT patients with UTUC underwent unilateral or bilateral nephroureterectomy, and subsequently, administered AC was rare. In the future, larger-scale collaborative studies and further molecular and genetic studies will be necessary. Second, unlike in most of the literature, AC was usually administered at UTUC stages ≥ T3. We applied AC for all UTUC patients (KT and non-KT patients) with stage ≥T2 as conducted in the POUT trial [7]. Furthermore, the aggressive characteristics of UTUC in immunosuppressant KT patients may prompt treating physicians to conduct AC at earlier stages. Third, the AC protocol was not standardized. However, most of the participants received three to four cycles of gemcitabine plus cisplatin or carboplatin. Fourth, we did not show all the histological subtypes of our resected surgical specimen. A retrospective study indicated that AC was only associated with an OS benefit in patients with pure UC [33]. However, AC should be considered when UC is the dominant pathology. Lastly, the operation method for the nephroureterectomy was determined by a treating surgeon according to preoperation image studies. Open-method surgeries may be considered in KT group patients whose UTUC is invasive or who have tissue scarring around graft vascularity. However, our study is still the first one to compare the oncological outcomes of locally advanced UTUC patients who underwent AC, specifically between KT and non-KT patients.

## 5. Conclusions

In our study, female sex, smaller tumor size, multifocal tumors, CIS, concurrent contralateral UTUC, and synchronous tumors in the renal pelvis and ureter were significantly more common in the KT group than the non-KT group. This also suggested that KT patients with UTUC stage ≥T2 who underwent AC after their surgeries presented worse OS and DFS rates compared to non-KT patients. KT patients tolerated the AC course well, and their nephrotoxicity was mild and acceptable.

## Figures and Tables

**Table 1:** Characteristics of enrolled patients.

	KT (*n* = 23)	Non-KT (*n* = 34)	*p* Value
Mean age (years)	59.39 ± 9.14	61.38 ± 10.23	0.455
Gender (M/F)	5/18	18/16	0.018
pT stage			0.159
pT2	6	4	
pT3	16	30	
pT4	1	0	
N stage			0.08
N0	21	34	
N1	0	0	
N2	2	0	
Tumor size (cm)	2.85 ± 2.78	4.93 ± 3.78	0.028
Margin positive/negative	4/19	4/30	0.549
Multifocality	17	14	0.015
History of contralateral UTUC (%)	11 (47.8)	1 (2.9)	<0.001
Previous	1 (4.3)	0 (0)	0.220
Concurrent	10 (43.5)	1 (2.9)	<0.001
Recurrent	2 (8.7)	0 (0)	0.08
History of bladder UC (%)	14 (60.9)	15(44.1)	0.215
Previous	5 (21.7)	2 (5.9)	0.074
Concurrent	8 (34.8)	5 (14.7)	0.076
Recurrent	10 (43.5)	12 (35.3)	0.533
High grade	22	32	0.799
CIS	10	6	0.033
LVI	10	12	0.533
Follow-up (months)	52.35 ± 34.56	64.71 ± 42.29	0.250
No. operative method (%) Laparoscopic Open	7 16	21 13	0.02
No. tumor location (%) Renal pelvis Ureter Synchronous renal pelvis + ureter	2 5 16	15 8 11	0.007
Chemotherapy cycle 1–2 3–6 ≥7 unknown	6 16 0 1	5 26 2 1	0.306
Adjuvant chemotherapy regimen G + C G + Carbo G MVAC unknown	13 10 0 0 0	18 11 2 2 1	0.366
Mean eGFR mL/min	65.1 ± 26.64	57.92 ± 23.26	0.285
No. eGFR ≥ 60 mL/min (%) No. eGFR < 60 mL/min (%)	12 (52.2) 11 (47.8)	20 (58.8) 14 (41.2)	0.620

UTUC, upper tract urothelial carcinoma; UC, urothelial carcinoma; CIS, carcinoma in situ; LVI, lymphovascular invasion; G + C, gemcitabine plus cisplatin; G + Carbo, gemcitabine plus carboplatin; MVAC, methotrexate, vinblastine, doxorubicin, and cisplatin; eGFR, estimated glomerular filtration rate.

**Table 2:** Univariate analysis and multivariate analysis using Cox proportional hazards regression model.

	Disease-Free Survival				Overall Survival			
	Univariate		Multivariate		Univariate		Multivariate	
	OR (95% CI)	*p* Value	OR (95% CI)	*p* Value	OR (95% CI)	*p* Value	OR (95% CI)	*p* Value
Kidney transplant	2.545 (1.159–5.590)	0.02	1.445 (0.575–3.629)	0.433	2.626 (1.031–6.687)	0.043	1.170 (0.400–3.422)	0.774
Age: <60 vs. ≥60	0.976 (0.445–2.142)	0.952			0.592 (0.233–1.506)	0.271		
Gender M vs. F	1.392 (0.639–3.031)	0.405			0.882 (0.350–2.221)	0.790		
pT stage: ≥pT3 vs. pT2	0.720 (0.270–1.916)	0.510			1.156 (0.335–3.990)	0.891		
N stage N2 vs. N0	2.378 (0.556–10.169)	0.243			1.097 (0.144–8.340)	0.929		
Tumor size: ≥4 vs. <4 cm	1.100 (0.513–2.358)	0.807			0.773 (0.314–1.906)	0.576		
Surgical margin: Positive vs. Negative	2.639 (1.092–6.378)	0.031	2.069 (0.815–5.250)	0.126	3.258 (1.233–8.605)	0.017	2.159 (0.765–6.092)	0.146
Multifocality	1.714 (0.768–3.827)	0.188			2.569 (0.926–7.129)	0.070		
Contralateral UTUC	1.693 (0.732–3.918)	0.219			1.728 (0.649–4.600)	0.273		
Bladder UC	1.320 (0.604–2.885)	0.486			0.970 (0.401–2.346)	0.946		
Tumor grade: High vs. Low	0.534 (0.123–2.305)	0.400			0.524 (0.120–2.293)	0.391		
CIS	0.811 (0.348–1.892)	0.628			0.777 (0.294–2.057)	0.612		
LVI	1.546 (0.716–3.340)	0.268			1.783 (0.734–4.332)	0.202		
Operative method: Open vs. Laparoscopy	5.057 (1.908–13.407)	0.001	3.783 (1.294–11.062)	0.015	4.044 (1.347–12.141)	0.013	3.466 (0.947–12.684)	0.060
Location		0.124		0.604		0.056		0.136
Renal pelvis vs. Ureter	0.281 (0.083–0.951)	0.041	0.540 (0.148–1.973)	0.351	0.071 (0.008–0.615)	0.016	0.100 (0.010–0.962)	0.046
Renal pelvis + ureter vs. Ureter	0.681 (0.284–1.633)	0.389	0.702 (0.264–1.867)	0.478	0.648 (0.240–1.752)	0.393	0.710 (0.233–2.163)	0.546
eGFR < 60 mL/min/1.73 m^2^	0.967 (0.443–2.110)	0.934			0.950 (0.382–2.365)	0.912		

M, male; F, female; UTUC, upper tract urothelial carcinoma; UC, urothelial carcinoma; CIS, carcinoma in situ; LVI, lymphovascular invasion; eGFR, estimated glomerular filtration rate.

**Table 3:** Drug toxicities in patients who received adjuvant chemotherapy.

	CTCAE	Kidney Transplant	Non-Kidney Transplant	*p* Value
Neutropenia	0 1–2 3–4	3 4 16	1 16 16	0.036
Anemia	0 1–2 3–4	0 12 11	5 23 5	0.01
Thrombocytopenia	0 1–2 3–4	3 10 10	10 18 5	0.047
Nausea/Vomiting	0 1–2 3	17 5 1	24 8 1	0.949
Nephrotoxicity	0 1–2 3–4	14 6 3	25 6 2	0.457
Hepatotoxicity	0 1–2 3–4	17 5 1	24 9 0	0.449
Skin rash	0 1–2	21 2	29 4	0.683

CTCAE, Common Terminology Criteria for Adverse Events.

## Data Availability

The data presented in this study are available on request from the corresponding author with a reasonable reason.
